# Supplementary material for: EB1 Restricts Breast Cancer Cell Invadopodia Formation and Matrix Proteolysis via FAK
Source: Cells. 2021 Feb 13;10(2):388. doi: 10.3390/cells10020388 (PMC7918453; doi:10.3390/cells10020388)
Supplement: Supplementary file 1 [file cells-10-00388-s001.pdf]

Supplementary Materials

EB1 restricts breast cancer cell invadopodia formation and matrix proteolysis via FAK.

Brice Chanez, Kevin Ostacolo, Ali Badache and Sylvie Thuault

Table S1: Sequences of siRNAs used in this study.

Table S2: Primer pairs used for amplifying FAK, FAK Y397F and EB1 sequences.

Figure S1: Knockdown efficiency of the siRNAs. MDA-MB-231 cells and MCF10A cells treated with TGF-β for 6 days were transfected with a control siRNA (siLacZ) or siRNAs against EB1 (siEB1_1 or siEB1_2), CLASP1 (siCLASP1), CLASP2 (siCLASP2) or CLIP170 (siCLIP170) and target protein expression levels assessed by western blotting using specific antibodies. α-Tubulin was used as loading control.

Figure S2: Foci are the consequence of MMP-dependent proteolytic activity. Representative images of MDA-MB-231 cells transfected with a control siRNA (siLacZ) or siRNAs against EB1 (siEB1_1 or siEB1_2) seeded on fluorescently-labeled gelatin (FITC-gelatin) and treated with the general inhibitor of metalloproteases GM6001 at 10μM for 4 hours. Cells were fixed and stained with an anti-Cortactin antibody to identify invadopodia.

Figure S3: Re-expression of EB1 reverts the increased degradative phenotype of EB1 depleted cells. MDA-MB-231 were co-transfected with a control siRNA (siLacZ) or the siEB1_2 targeting the 3'UTR of EB1 mRNA and a plasmid expressing the mCherry alone or EB1 fused to mCherry before seeding on fluorescently-labeled gelatin (FITC-gelatin) for 4 hours. (A) Levels of endogenous EB1 and EB1 fused to mCherry were analyzed by western blotting (WB) using EB1 antibody. α-Tubulin serves as a loading control. (B) Representative images of cells fixed and stained with anti-mCherry and anti-Cortactin antibodies are shown. The degraded area (C) and the number of degradation foci (D) per mCherry positive degrading cells are represented as the mean ± SEM of three independent experiments. Mean of each individual experiment is reported. Statistical analysis was performed as described in Figure 1. *** $p \leq 0.001$, ** $p \leq 0.01$, * $p \leq 0.05$. Full-length blots are presented in Supplementary Figure S6.

Figure S4: FAK overexpression inhibits ECM proteolysis. MDA-MB-231 cells stably expressing mCherrry, mCherry fused to wild type FAK or mCherry fused to the SH2-binding motif mutant of FAK (FAK Y397F) were transfected with a control siRNA (siLacZ) before seeding on fluorescently-labeled gelatin (FITC-gelatin) for 4 hours. Representative images of cells fixed and stained with anti-mCherry and anti-Cortactin (not show) antibodies. The percentage of mCherry positive degrading cells (B), the degraded area per cell (C) and the number of degradation foci (D) are represented as the mean ± SEM of three independent experiments. Mean of each individual experiment is reported. Statistical analysis was performed as described in Figure 1. *** $p \leq 0.001$, ** $p \leq 0.01$, * $p \leq 0.05$.

Figure S5: Microtubule targeting agents do not impair ECM degradation, nor FAK activation levels. MDA-MB-231 cells treated at the time of seeding with the indicated drugs were seeded on gelatin-coated coverslips for 4 hours. (A) Representative images of cells fixed and stained with anti-α-Tubulin and anti-EB1 antibodies are shown. The white-boxed regions are shown enlarged in the

corner. Scale bars represent 10μm in non-enlarged images, 4μm in enlarged images. (B) Representative images of cells fixed and stained with anti-Cortactin and anti-TKS5 antibodies to quantify invadopodia, as in Figure 1, are shown. The ability of cells to degrade fluorescently-labeled gelatin was analyzed. The degraded area per cell (C) and the number of degradation foci (D) are represented as the mean ± SEM of three independent experiments. Statistical analysis was performed as described in Figure 1. (E) Levels of FAK and FAK phosphorylated on Tyr397 (p-Y397-FAK) were analyzed by western blotting using specific antibodies. α-Tubulin serves as a loading control. The ratio p-Y397-FAK:total FAK is represented as the mean ± SEM of three independent experiments. The paired *t* test was used to determine significant differences. ns not significant.

Figure S6: Uncropped western blots.

Table S1: Sequences of siRNAs used in this study.

Gene	NCBI Reference Sequence	Sequence
siLacZ	M55068	GCGGCUGCCGGAAUUUACCTT
siEB1_1	NM_012325	UUAAAUACUCUUAAGGCAUTT
siEB1_2	NM_012325	GAAUUGAAUUUUAAGCUAATT
siAPC_1	NM_000038	UUAUUCUUAAUUCCACAUCTT
siAPC_2	NM_000038	UUAUGCUCAAUGCUUAGUCTG
siACF7	NM_012090	UAACCUUCUGAUAAGAUUGTT
siCLASP1	NM_015282	UAUUGCUGCAUCUCGAACCTG
siCLASP2	NM_015097	UUCUCGACUAGAAUAAAUCTG
siCLIP170	NM_002956	UUUCUUUGUAUGUCAGAGCTG

Table S2: Primer pairs used for amplifying FAK, FAK Y397F and EB1 sequences.

Name	Sequence
FAK_For_GW	GGGGACAAGTTTGTACAAAAAAGCAGGCTTCATGGCAGCTGCTTACCTTGACCCCA
FAK_FL_rev_GW	GGGGACCACTTTGTACAAGAAAGCTGGGTCTCAGTGTGGTCTCGTCTGCCCTAGC
attB1-Nter EB1	GGGGACAAGTTTGTACAAAAAAGCAGGCTTCGAAGGAGATAGAACCATGGCAGTG AACGTATACTCAACGTCAG
attB2-Cter EB1 FL	GGGGACCACTTTGTACAAGAAAGCTGGGTCATACTCTTCTTGCTCCTCCTGTGGGC

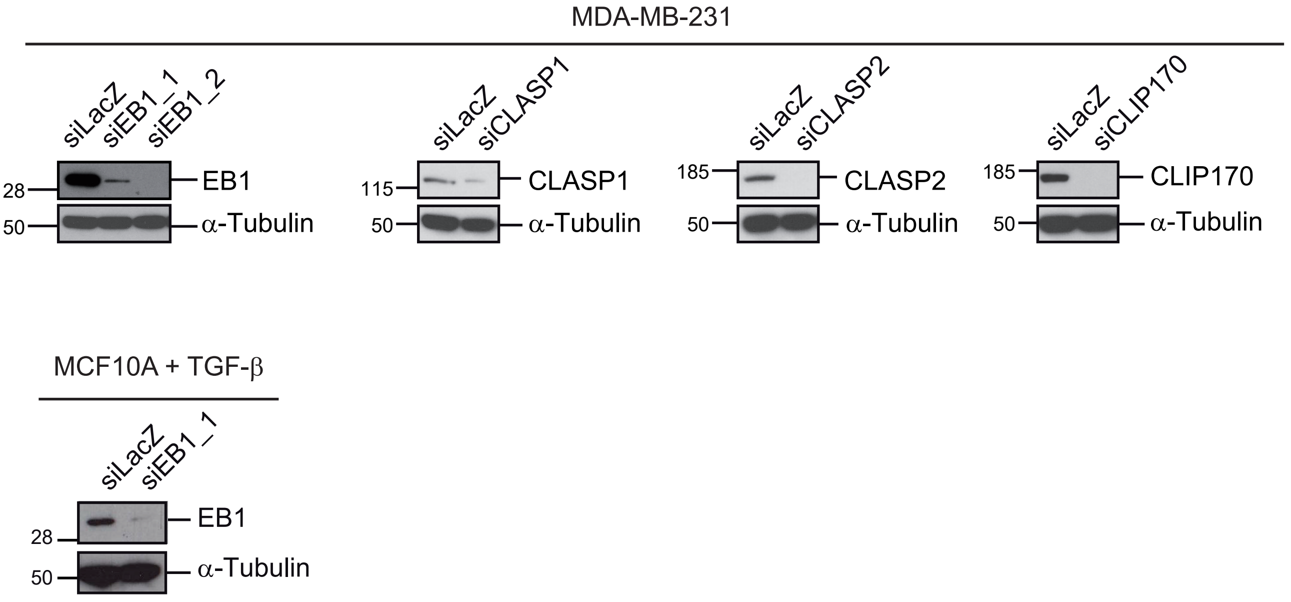

Chanez et al., Figure S1

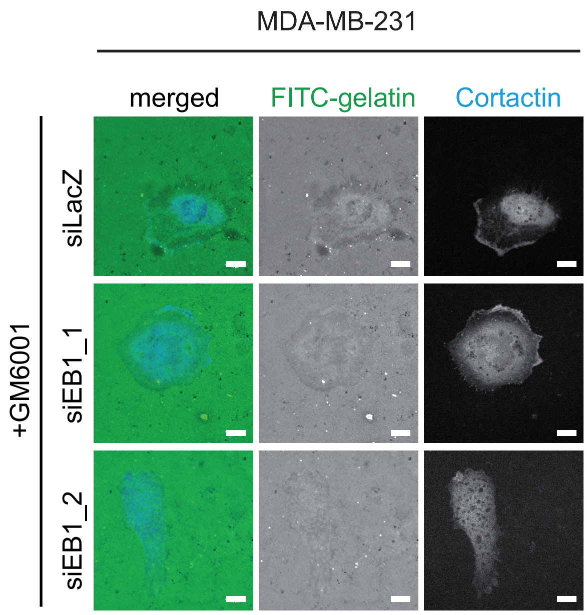

Chanez et al., Figure S2

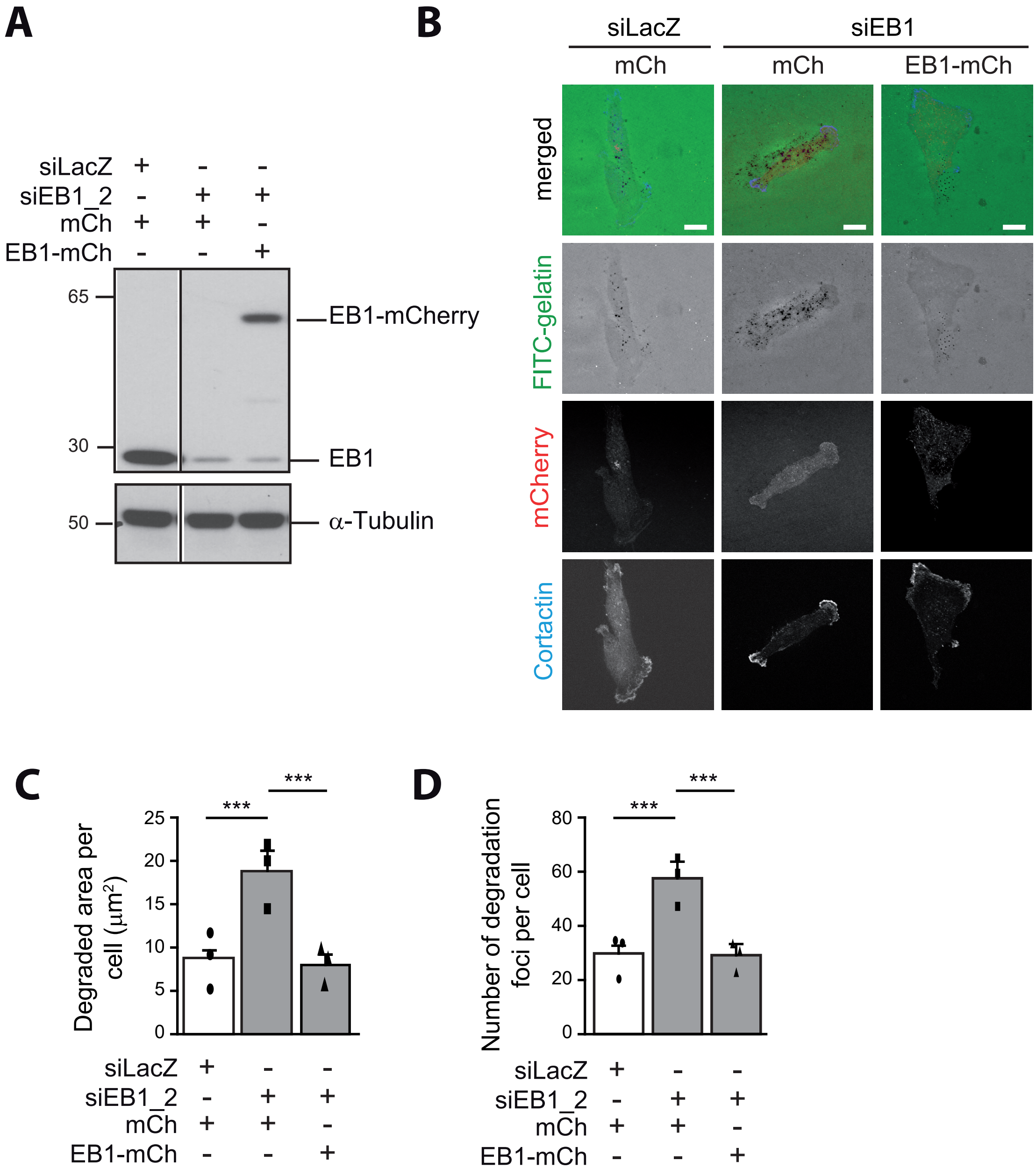

Chanez et al., Figure S3

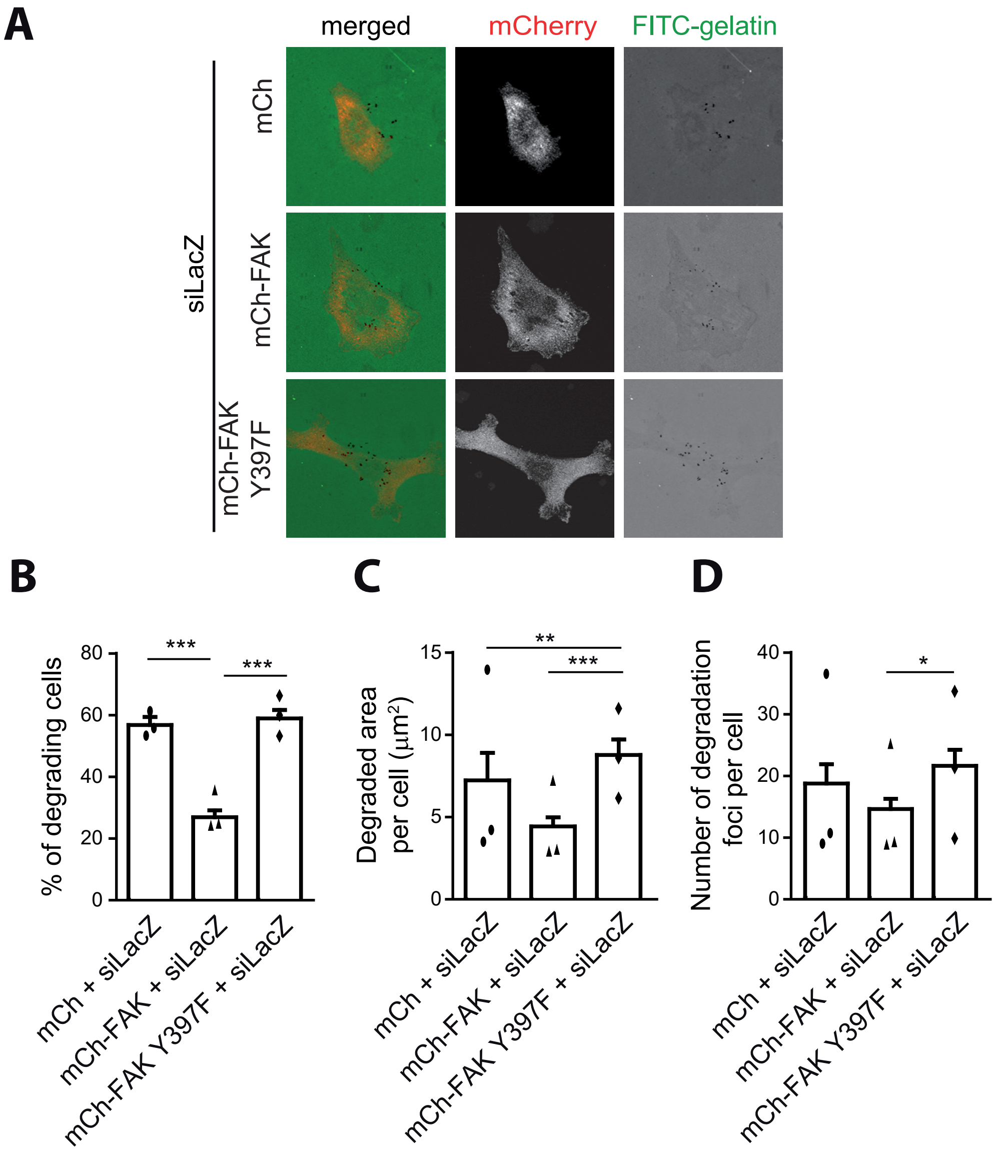

Chanez et al., Figure S4

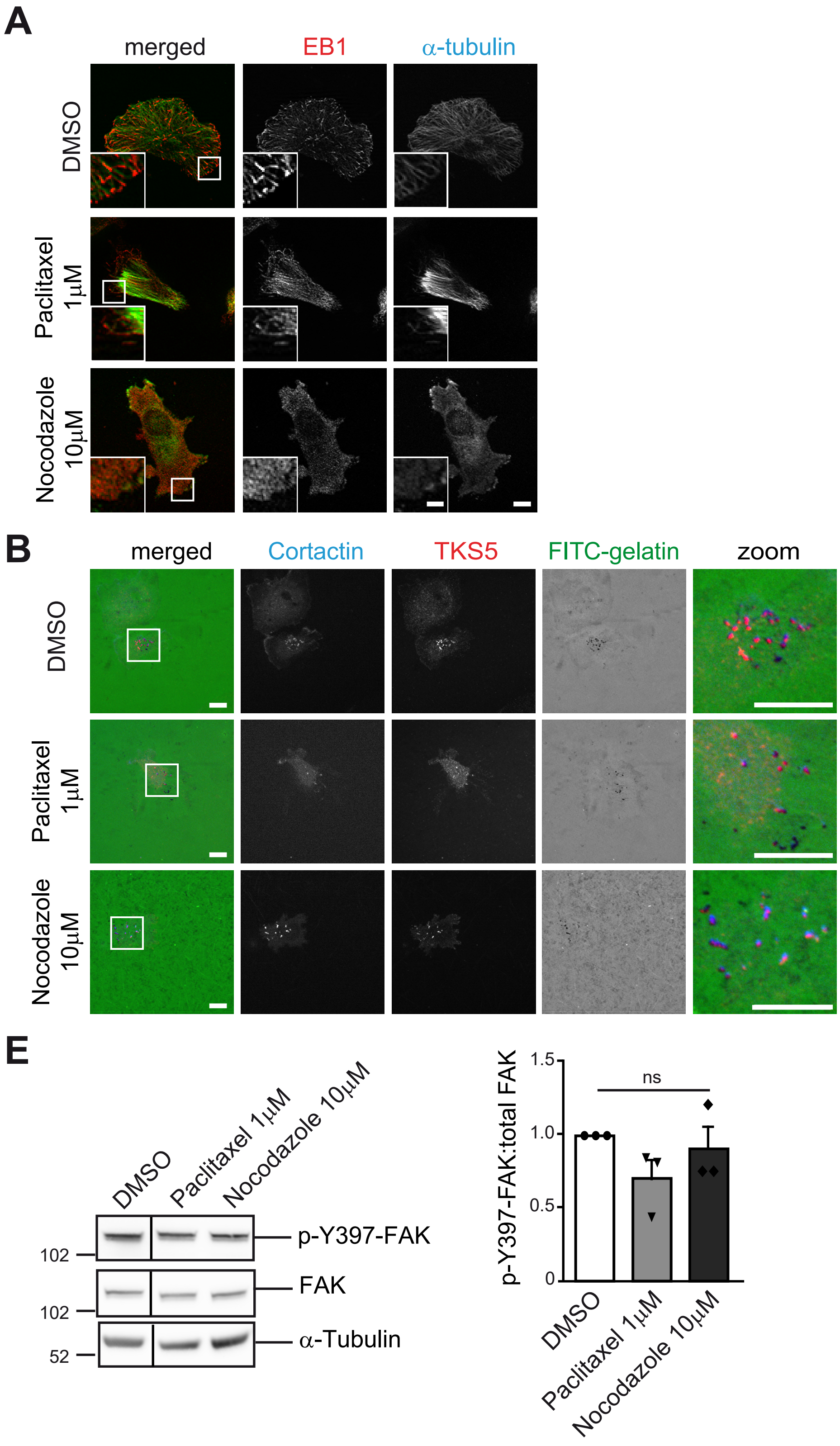

Chanez et al., Figure S5

Figure 4A

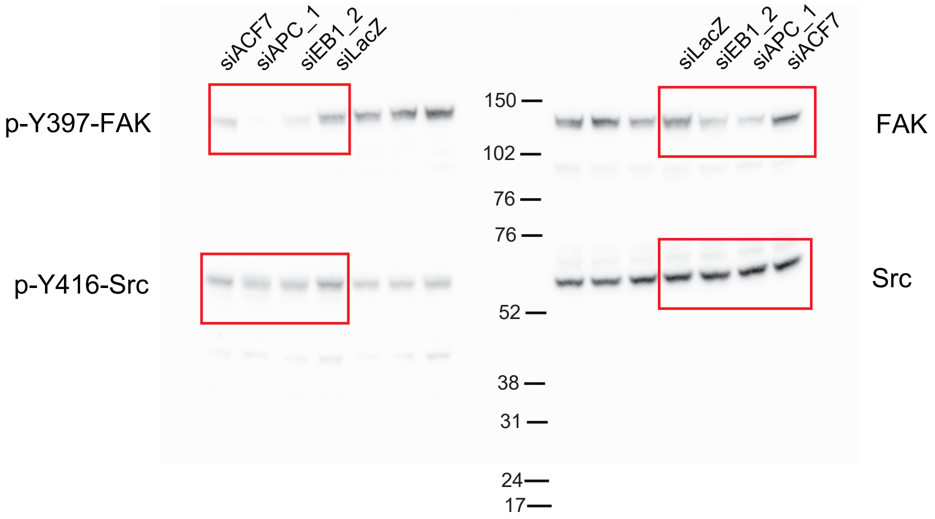

Figure 4C

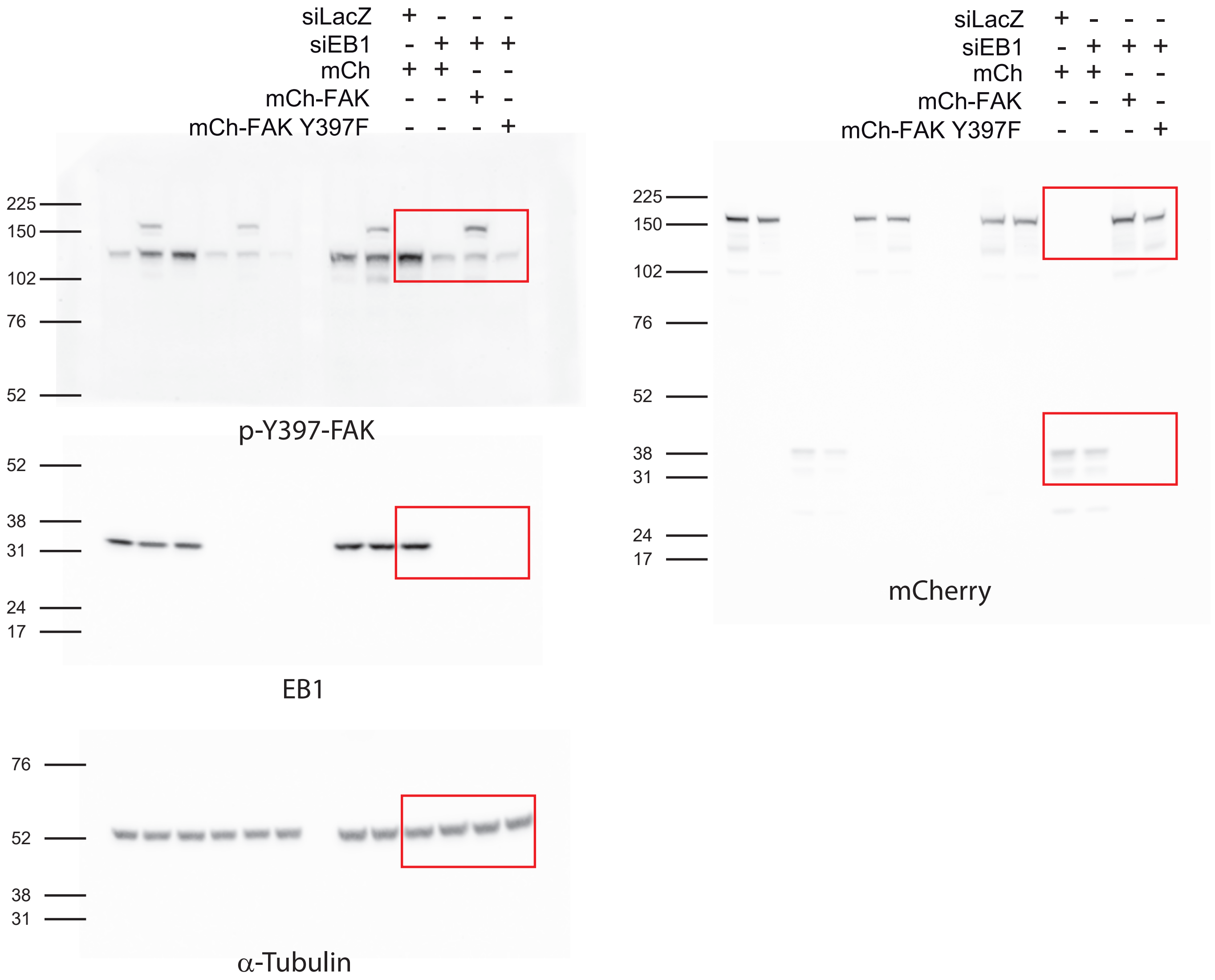

Chanez et al., Figure S6

Figure S1

MDA-MB-231

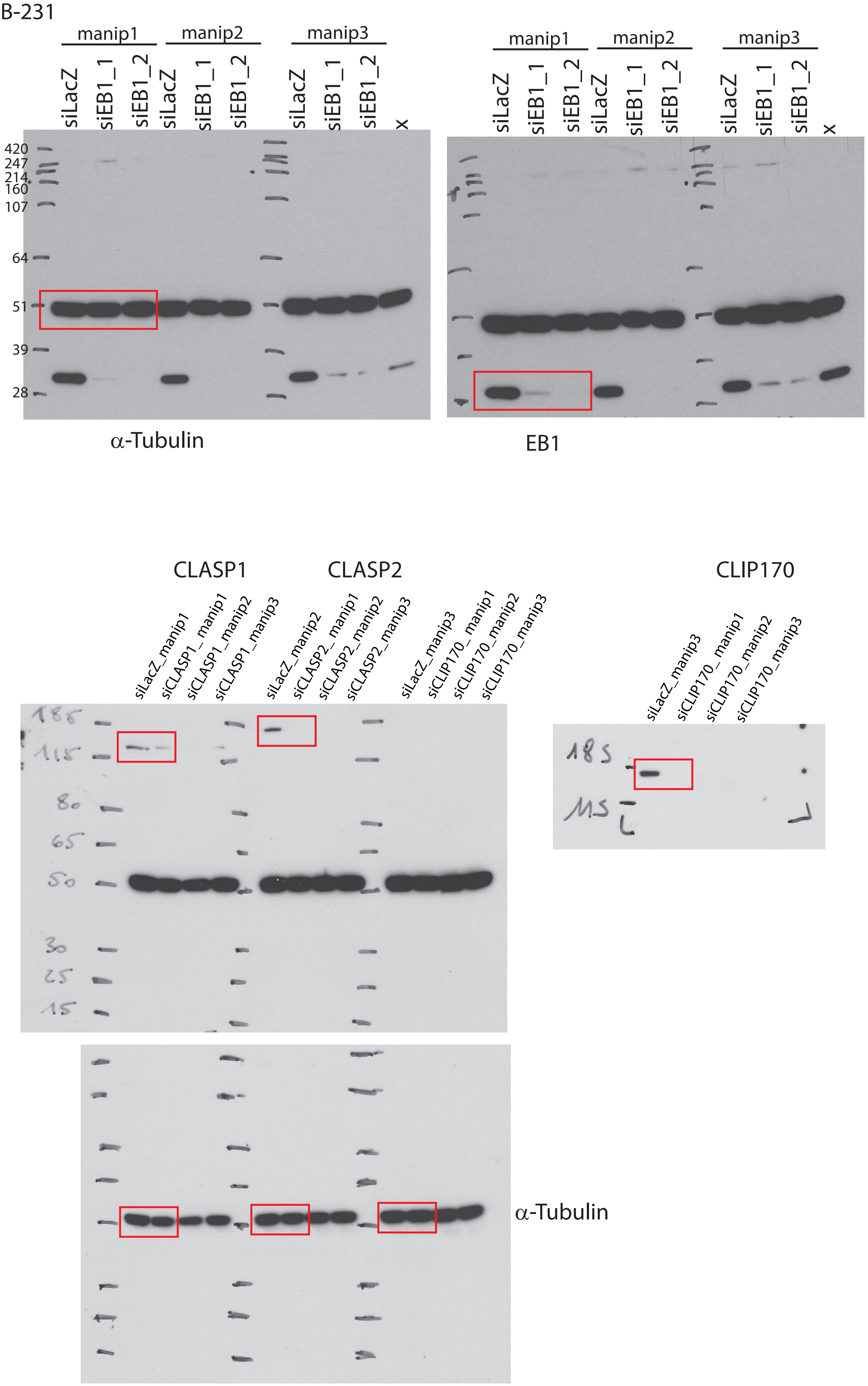

Chanez et al., Figure S6 continued

Figure S1

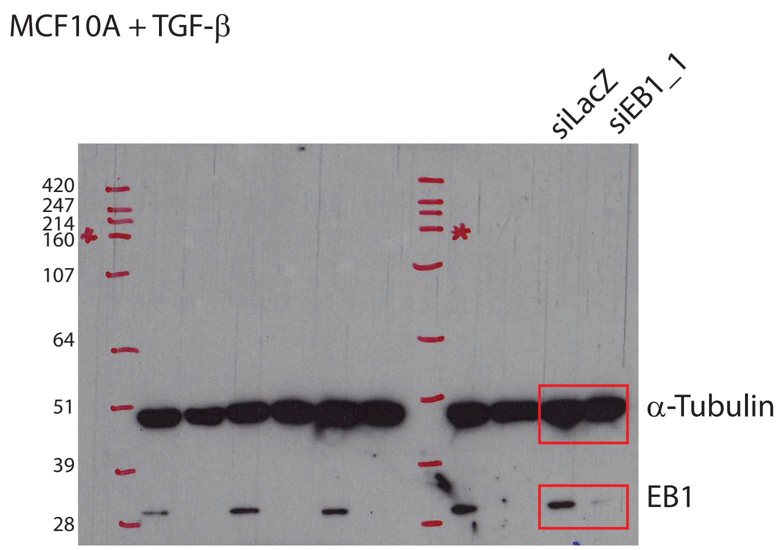

Figure S3

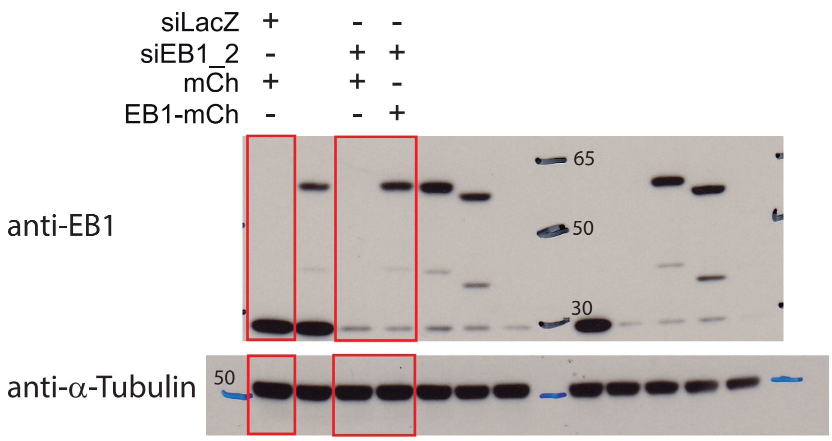

Figure S4E

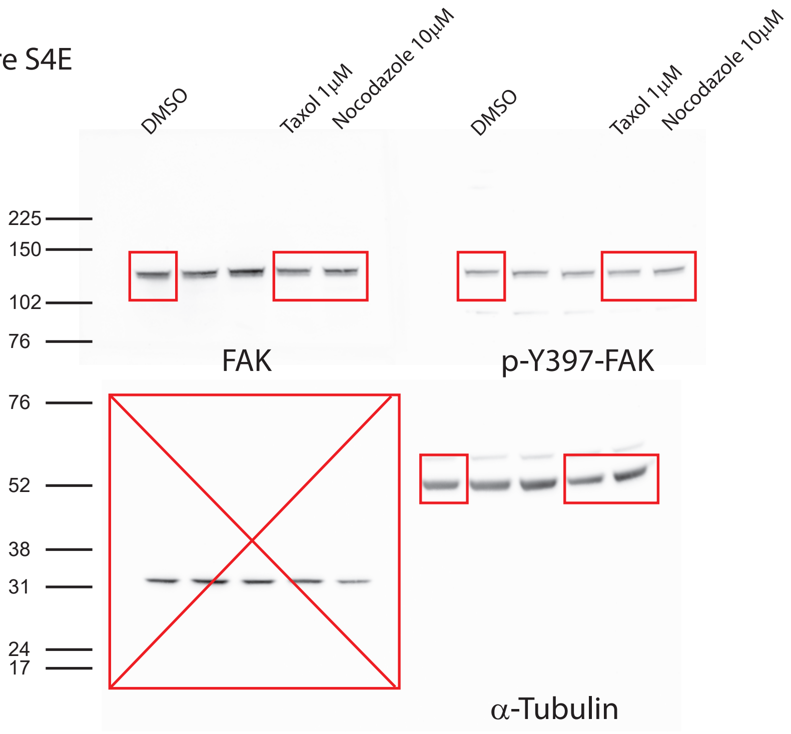

Chanez et al., Figure S6 continued
